# Quantification of age-related changes in the structure and mechanical function of skin with multiscale imaging

**DOI:** 10.1007/s11357-024-01199-9

**Published:** 2024-05-18

**Authors:** Alan E. Woessner, Nathan J. Witt, Jake D. Jones, Edward A. Sander, Kyle P. Quinn

**Affiliations:** 1 Department of Biomedical Engineering, University of Arkansas, 123 John A. White Jr. Engineering Hall, Fayetteville, AR 72701 USA; 2 Arkansas Integrative Metabolic Research Center, University of Arkansas, Fayetteville, AR USA; 3 Roy J. Carver Department of Biomedical Engineering, University of Iowa, Iowa City, IA USA

**Keywords:** Skin, Intrinsic Aging, Biomechanics, Multi-scale, Second Harmonic Generation, Multiphoton Microscopy

## Abstract

The mechanical properties of skin change during aging but the relationships between structure and mechanical function remain poorly understood. Previous work has shown that young skin exhibits a substantial decrease in tissue volume, a large macro-scale Poisson’s ratio, and an increase in micro-scale collagen fiber alignment during mechanical stretch. In this study, label-free multiphoton microscopy was used to quantify how the microstructure and fiber kinematics of aged mouse skin affect its mechanical function. In an unloaded state, aged skin was found to have less collagen alignment and more non-enzymatic collagen fiber crosslinks. Skin samples were then loaded in uniaxial tension and aged skin exhibited a lower mechanical stiffness compared to young skin. Aged tissue also demonstrated less volume reduction and a lower macro-scale Poisson’s ratio at 10% uniaxial strain, but not at 20% strain. The magnitude of 3D fiber realignment in the direction of loading was not different between age groups, and the amount of realignment in young and aged skin was less than expected based on theoretical fiber kinematics affine to the local deformation. These findings provide key insights on how the collagen fiber microstructure changes with age, and how those changes affect the mechanical function of skin, findings which may help guide wound healing or anti-aging treatments.

## Introduction

Skin serves primarily as a protective barrier to the environment, but the structural and functional properties of each layer of our skin decline with advanced age. The outermost epidermis is a semi-permeable layer between the body and its environment [1]. During the natural process of aging, the epidermal layer becomes thinner due to flattening of the dermal–epidermal junction [2, 3]. The middle dermal layer acts as the primary load bearing structure for skin, and it consists of a heterogeneous 3D extracellular network of interconnected collagen and elastin fibers, often described as a dense “basket-weave” structure [2, 4]. With increased age, the dermis becomes less hydrated due to a reduction in glycoprotein content [4, 5]. In addition, a reduction in the rate of collagen turnover results in a decrease in the number of collagen fibers and the connections between them, as well as an increased amount of non-enzymatic crosslink fragments dispersed within the collagen fiber matrix [5]. Finally, the innermost hypodermis acts as a thermoregulator and site for fatty energy storage, but the amount of fat contained in the hypodermis decreases with age [6]. Collectively, these changes in skin structure affect its mechanical function and lead to an increase in skin fragility [7–9], resulting in higher rates of injury, poorer healing outcomes, and heightened co-morbidities. However, the complex and multiscale relationships that exist between skin structure and function need to be better understood so that new approaches to maintain or improve skin health can be developed.

During intrinsic aging, there are many physical and chemical changes that occur within skin such as increased wrinkling, changes to pH, and an altered dermal microstructure [2, 10]. Historically, histological staining of thin tissue sections has been performed to link an altered microstructure to changes in the biomechanical properties of aged skin [11–13]. This standard technique, however, is destructive and does not allow for an understanding of how the skin microstructure responds to mechanical loading. In particular, quantifying collagen fiber kinematics in response to mechanical loading is necessary for understanding how the mechanical behavior of skin relates to the organization and heterogeneity of the microstructure. Advanced multi-scale computational models can be used to obtain a better understanding of the relationships between mechanical function and tissue microstructure [14], but there is limited experimental work that includes multiscale kinematic and kinetic data to challenge and validate these computational models. Optical techniques, such as small angle light scattering and quantitative polarized light imaging, have been used to infer 2D fiber kinematics during stretching of various tissues and tissue substitutes, such as porcine aortic valve, human facet capsular ligaments, and collagen gels [15–18]. Additionally, previous studies that utilized 2D digital image correlation of mouse and porcine skin deformation during uniaxial tensile loading revealed that collagen fiber kinematics have a non-affine relationship with local tissue deformation [19, 20]. Skin has also been found to demonstrate a large Poisson’s ratio, indicating that there is substantial compaction in the direction orthogonal to imposed stretch [21–23]. Overall, these studies have provided key initial insights into the relationship between skin structure and function despite an inability to resolve individual collagen fibers and their 3D organization [16, 17, 24, 25].

Depth-resolved optical microscopy techniques, such as reflectance confocal microscopy, have been used to assess 3D collagen fiber kinematics during mechanical loading in collagenous gels [26, 27]. However, reflectance confocal microscopy lacks the ability to image collagen fiber organization deep within skin due to the effects of light scattering. Alternatively, multiphoton microscopy is a non-destructive depth-resolved imaging technique that can be used to visualize collagen fibers much deeper within tissue via second harmonic generation (SHG), and can provide information on non-enzymatic collagen crosslinking with two-photon excited fluorescence (TPEF) [20, 28, 29]. SHG microscopy has been previously used in conjunction with mechanical testing to characterize the 2D collagen fiber kinematics of mouse skin, as well as quantify age-related differences in tissue kinematics and collagen fiber alignment during loading [30]. Despite the 3D imaging capabilities of SHG microscopy, these previous studies have only focused on 2D assessments of fiber responses within the skin microstructure.

Recently, we have combined SHG microscopy with mechanical stretching to quantify the 3D microstructural tissue and fiber kinematics of young mouse skin. Our work revealed a substantial decrease in tissue volume at multiple length-scales during uniaxial tensile stretching, but similar analysis has not been performed on aged skin [23]. Building from that foundational study, the goal of this current study was to identify differences in the 3D microstructure and mechanical function between young and aged skin. Incremental uniaxial tensile loading of young and aged mouse skin was performed with a uniaxial mechanical testing device integrated with a multiphoton microscope and wide field camera. Microstructural organization was quantified and the magnitude of realignment during mechanical stretching was assessed. Additionally, 3D collagen fiber features at the micro-scale and 2D fiduciary markers at the macro-scale were tracked and used to quantify age-related changes in tissue kinematics across multiple length scales. Quantification of age-related changes in the relationship between microstructure and mechanical response will provide key insights for understanding the complex and dynamic nature of skin.

## Methods and materials

### Multi-scale data collection

Ventral skin from young, 4 month old (~ 25% life span; n = 6) [23] and aged, 23 month old (~ 75% life span; n = 6) C57BL/6 J mice was excised and flash frozen until mechanical testing [31]. Based on previous literature probing the effect of flash freezing on rat tendon, this approach to storing tissue samples should not change its microstructure or mechanical properties [32]. Prior to testing, skin was prepared by cleaning the epidermis and carefully resecting the hypodermis. As in previous studies, traditional dog-bone shapes with a 7.11 mm × 2.03 mm gauge region were cut from the tissue using a punch, and fiduciary dots were marked on the tissue using a fine-tipped pen [33]. The tissue was then placed into a mechanical tester (ADMET; Norwood, MA) integrated with a multiphoton microscope (Bruker; Billerica, MA) equipped with a wide-field camera (Lumenera; Ottawa, ON), enabling 3D micro-scale and 2D macro-scale imaging of the tissue during mechanical testing [23]. For the duration of mechanical testing, the sample was submerged in phosphate-buffered saline (PBS).

The tissue geometry, including thickness, was measured using the wide-field camera imaging system, and then the tissue was pre-stressed to 10 kPa followed by preconditioning to ~ 5% strain for 20 cycles (4 mm/min; ~ 20%/min strain rate) [23]. After preconditioning, SHG (855 nm ex./427 nm em.) and TPEF (755 nm ex./460 nm em.) were measured for three image volumes (512 × 512 × 130 pixels; 1.144 × 1.144 µm/pixel resolution; 1 µm z-step; ~ 2.5 min/image volume) spanning the gauge region of the tissue. The location of the second field of view, which is in the center of the gauge region, was noted and tracked during the mechanical test to collect the same field-of-view during the mechanical test. Mechanical testing was performed in the lateral direction in 1 mm displacement increments at a quasi-static rate of 0.2 mm/min until mechanical failure occurred. During stretching, mechanical force was collected with a 100 N load cell at a rate of 100 Hz, and macro-scale images (2464 × 2056 pixels; 0.02 mm/pixel resolution) were collected at a rate of 0.2 Hz. At each 1 mm increment of displacement, high-resolution 3D micro-scale SHG images were collected for the same field of view located in the center of the gauge region.

### Macro-scale and mechanical data analysis

**Fig. 1:**
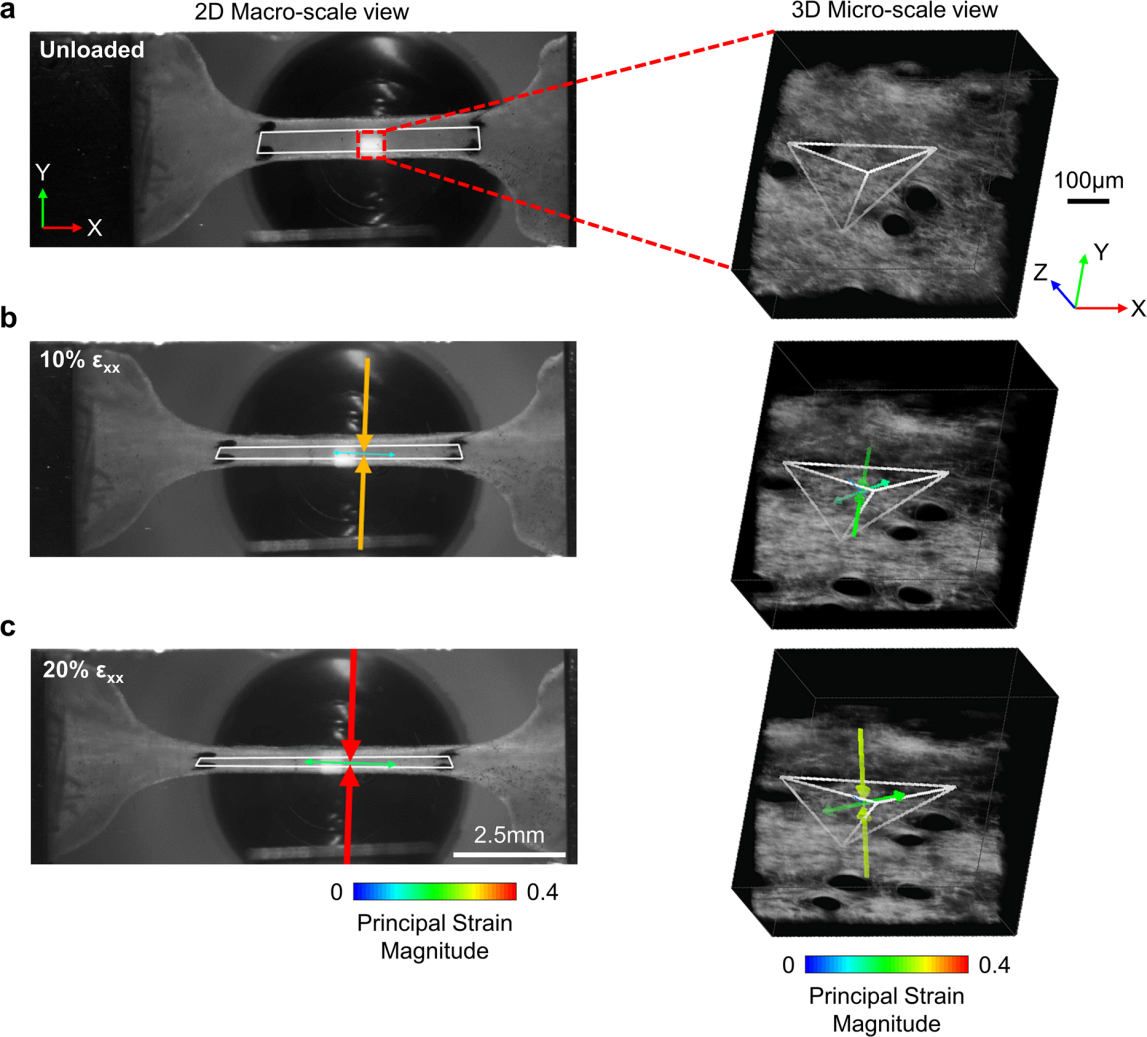
Based on a reference unloaded configuration at both the 2D macro-scale (**A; left**) and 3D micro-scale (**A; right**), multiscale tissue kinematics were quantified at 10% (**B**) and 20% (**C**) Green strain in the direction of loading (ε_xx_). The representative images shown were taken from young mouse skin. The white polygons correspond to tracked features and arrows represent the magnitude and direction of principal strains

Strain from macro-scale images of the tissue sample were analyzed as previously described [23]. Briefly, fiducial markers on the tissue were tracked during the mechanical test via an automated normalized cross-correlation algorithm. After tracking the location of each marker, isoparametric mapping of the marker locations was used to calculate the 2D deformation gradient (*F*) and Green strain (ε) tensor of the tissue. Measured loads were converted to engineering stress by normalizing based on the optically measured undeformed cross-sectional area of the tissue. At each increment of displacement starting at 1 mm, incremental Young’s moduli (*E*_*i*_) were calculated using a backward difference approximation,

$${E}_{i}= {(\sigma }_{i}-{\sigma }_{i-1})/({\varepsilon }_{xx,i}-{\varepsilon }_{xx,i-1})$$

where σ is the engineering stress at the current increment *i* and ε_xx_ is the macro-scale Green strain in the direction of stretch [16]. This incremental measure of Young’s modulus allows for the material properties of the tissue to be quantified when the tissue is in static equilibrium. Principal strain magnitudes and directions were computed from the Green strain tensor (Fig. 1, left), as well as an incremental Poisson’s ratio measurement ν_*i*_,

$${\nu }_{i}= -{\varepsilon }_{yy,i}/{\varepsilon }_{xx,i}$$

where ε_yy_ is the Green strain perpendicular to the direction of loading.

During mechanical loading, it was noted that each tissue sample, regardless of age group, mechanically failed at strains greater than 20% ε_xx_ (i.e. Green strain along the loading axis). Therefore, to quantify how the microstructure is affected by different magnitudes of stretch, fiber kinematics were specifically tabulated at three magnitudes of stretch: 10% ε_xx_, 20% ε_xx_, and at the maximum Young’s modulus which typically corresponded to the increment prior to failure. These three values roughly correspond to the mechanical behavior of tissue within the toe/heel region (10% ε_xx_), toe/heel to linear transition region (20% ε_xx_), and at failure [2].

### Micro-scale data analysis

**Fig. 2:**
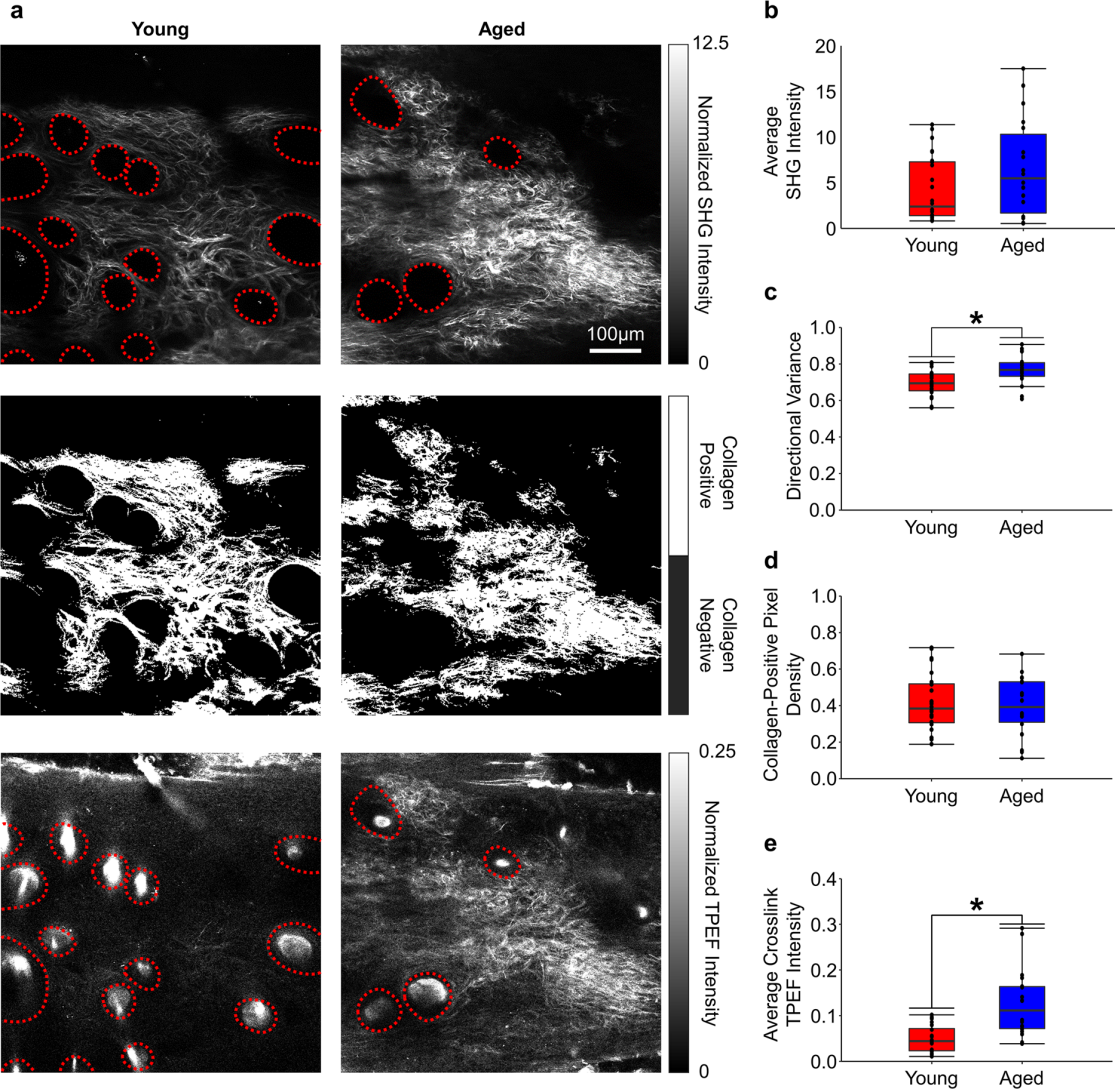
SHG and TPEF images of the undeformed microstructure of young (**A; left**) and aged (**A; right**) skin allow for visualization of different dermal structures, including dermal collagen (via SHG), hair follicles (via TPEF; red circles), and collagen crosslink autofluorescence (via TPEF). Using the SHG images (**A; top**), a trained CNN segmented collagen-positive pixels (**A; middle**), and collagen-positive masks were applied to SHG and TPEF (**A; bottom**) images. Within collagen-positive regions, the average collagen SHG intensity (**B**), collagen fiber directional variance (**C**), collagen-positive pixel density (**D**), and the average collagen crosslink TPEF intensity (**E**) were quantified. Although there was no significant difference in the intensity of the collagen SHG signal (**B**) or collagen-positive pixel density (**D**) with increased age, the fibers in aged tissue showed significantly higher non-enzymatic crosslink fluorescence (**E**) and were more randomly organized (**C**). All measurements were made in the undeformed configuration. N = 6 for both age groups, * represents p < 0.05

Prior to image analysis, the collagen-containing pixels within an image volume were determined by inputting all 2D SHG image slices into a previously trained convolutional neural network (CNN) capable of accurately classifying collagen-positive pixels within an image [34]. Masks of collagen-positive pixels were then recombined to form a 3D mask of collagen-positive pixels within an image volume. For each image volume of both TPEF and SHG signal, the collected intensities were normalized to a known concentration of fluorescein based on the laser power and detector gain [28, 35]. To quantify the collagen-containing pixel percentage, a tissue-positive mask was also generated by thresholding a summed normalized intensity volume from all emission channels with the overall average intensity. For image volumes that were acquired prior to mechanical loading, the number and percentage of collagen-positive pixels, their average collagen SHG intensity, and their average collagen crosslink TPEF intensity within collagen-positive pixels were calculated and compared between age groups (Fig. 2). Within each image volume, 3D collagen fiber orientation was measured by measuring pixel-wise fiber orientation in the X–Y plane (θ) and inclination out of the plane (φ) using a 3D vector-weighted summation algorithm within a 7 × 7 × 7 pixel window [36]. To quantify the magnitude of overall fiber alignment, 3D directional variance, which ranges between 0 (completely aligned) and 1 (completely random), was calculated from all collagen-positive pixels within an image volume [23, 36]. The amount of fiber re-alignment during loading was then quantified as the change in directional variance between the initial undeformed configuration and three points along the stress–strain curve: 10% and 20% micro-scale Green strain, and the strain at the maximum recorded Young’s modulus.

To compute micro-scale tissue strain tensors, 3D SHG volumes collected in the center of the gauge region during stretch were first registered to the initial image volume using a 3D Fourier-based approach [37]. Collagen SHG features (20 × 20 × 5 pixels) for tracking were determined from the initial image volume using a 3D corner-based algorithm, and the initial positions (*x*_*0*_, *y*_*0*_, *z*_*0*_) for each feature were logged. At each increment of displacement, the new positions of each feature (*x*_*i*_*, y*_*i*_*, z*_*i*_) were computed from a local window (20 × 20 × 20 pixels) using a 3D normalized cross-correlation algorithm [23]. To increase the precision of the feature tracking, the SHG feature at the current position (*x*_*i*_, *y*_*i*_, *z*_*i*_) were then tracked to the previous increment (*i-1*), and the position (*x*’_*i-1*_, *y*’_*i-1*_, *z*’_*i-1*_) was logged. The amount of incremental tracking error was calculated as the Euclidian distance between points (*x*’_*i-1*_, *y*’_*i-1*_, *z*’_*i-1*_) and (*x*_*i-1*_, *y*_*i-1,*_* z*_*i-1*_) [38]. After tracking SHG features through each increment to the final increment (*n*), feature positions (*x*_*n*_, *y*_*n*_, *z*_*n*_) were used to generate new SHG features from the final image volume. The newly generated features were then similarly tracked backward through each increment, resulting in a final (*x*’_*0*_, *y*’_*0*_, *z*’_*0*_) position for each feature. The Euclidian distance was calculated between (*x*_*0*_, *y*_*0*_, *z*_*0*_) and (*x*’_*0*_, *y*’_*0*_, *z*’_*0*_) to assess the amount of overall tracking error. The criterion for features that tracked well was defined as having a maximum of < 10 pixels of incremental tracking error, and < 1 pixel overall tracking error. From all the well-tracked points, the largest possible tetrahedron was determined and used to compute an incremental 3D deformation gradient (*F*), and Green strain tensor (ε).

The micro-scale Green strain tensor was used to calculate principal strain directions and magnitudes (Fig. 1, right), and the determinant of the deformation gradient was used to calculate a volume ratio (V/V_0_),

$$V/{V}_{0}=det(F).$$

Similar to the macro-scale and mechanical data analysis, the volume ratio measured at 10% and 20% micro-scale Green strain (ε_xx_) was compared between age groups to characterize the tissue kinematics at multiple time points throughout the mechanical test.

Finally, the relationship between fiber realignment and local tissue deformation was assessed. For each tissue sample, the 3D deformation gradients at 10% and 20% micro-scale Green strain (ε_xx_), as well as the strain at the maximum Young’s modulus measurement were applied to the initial image volume in a theoretically affine manner using built-in MATLAB functions (*affine3D.m* and *imwarp.m*). Within the theoretically deformed image volumes, the 3D fiber orientation and 3D directional variance was computed as described above, and the difference between measured and theoretically affine 3D directional variance was compared [23].

### Statistical analysis and data visualization

Since three image volumes spanning each sample were used to compare the initial micro-scale 3D directional variance, TPEF and SHG intensity, and number of collagen-positive pixels between age groups, one-way ANOVAs were used where each tissue sample was a nested random variable. All macro-scale and micro-scale kinematics, as well as the mechanical response at 10% strain, 20% strain, and maximum Young’s modulus were compared between age groups with unpaired students t-tests. Finally, the change in measured and theoretically affine 3D directional variance was compared to an affine response (ΔDV = 0) with a one-sample t-test. Significant differences were defined as a p-value < 0.05, and all statistical comparisons were performed in R (R Core Team; Vienna, Austria). For data visualization, box and whisker plots were generated within R (R Core Team; Vienna, Austria) using the *ggplot2* package [39]. Box and whisker plots were generated such that boxes represent the interquartile range (IQR), whiskers represent the maximum and minimum values, and outliers were considered as values that extended beyond 1.5*IQR.

## Results

### Undeformed tissue microstructure

Prior to mechanical loading, microstructural differences between young and aged skin were noted from the SHG and TPEF images. The images revealed that the SHG intensity of the dermal collagen fibers was similar in young and aged skin, but the TPEF intensity of the collagen fibers was noticeably brighter in aged skin (Fig. 2A). Within the collagen-positive regions defined by the trained CNN, the average normalized SHG intensity was computed and confirmed that no significant difference (p = 0.37) between the young and aged skin was detected, suggesting age does not affect fiber size (Fig. 2B). The 3D directional variance of collagen fibers in aged tissue (0.77 ± 0.08) was significantly higher compared to young tissue (0.69 ± 0.07; p = 0.01), indicating less aligned fibers with advanced age (Fig. 2C). The percentage of collagen-positive pixels in young tissue (41.61 ± 15.89%) and aged tissue (39.58 ± 16.39%), was not significantly different (p = 0.79) (Fig. 2D). However, the average normalized TPEF intensity within collagen-positive pixels of aged tissue (0.13 ± 0.07) was significantly brighter than young tissue (0.05 ± 0.03; p = 0.02), indicating an accumulation of non-enzymatic crosslinks with advanced age (Fig. 2E) [28].

### Macro-scale tissue response during loading

**Fig. 3:**
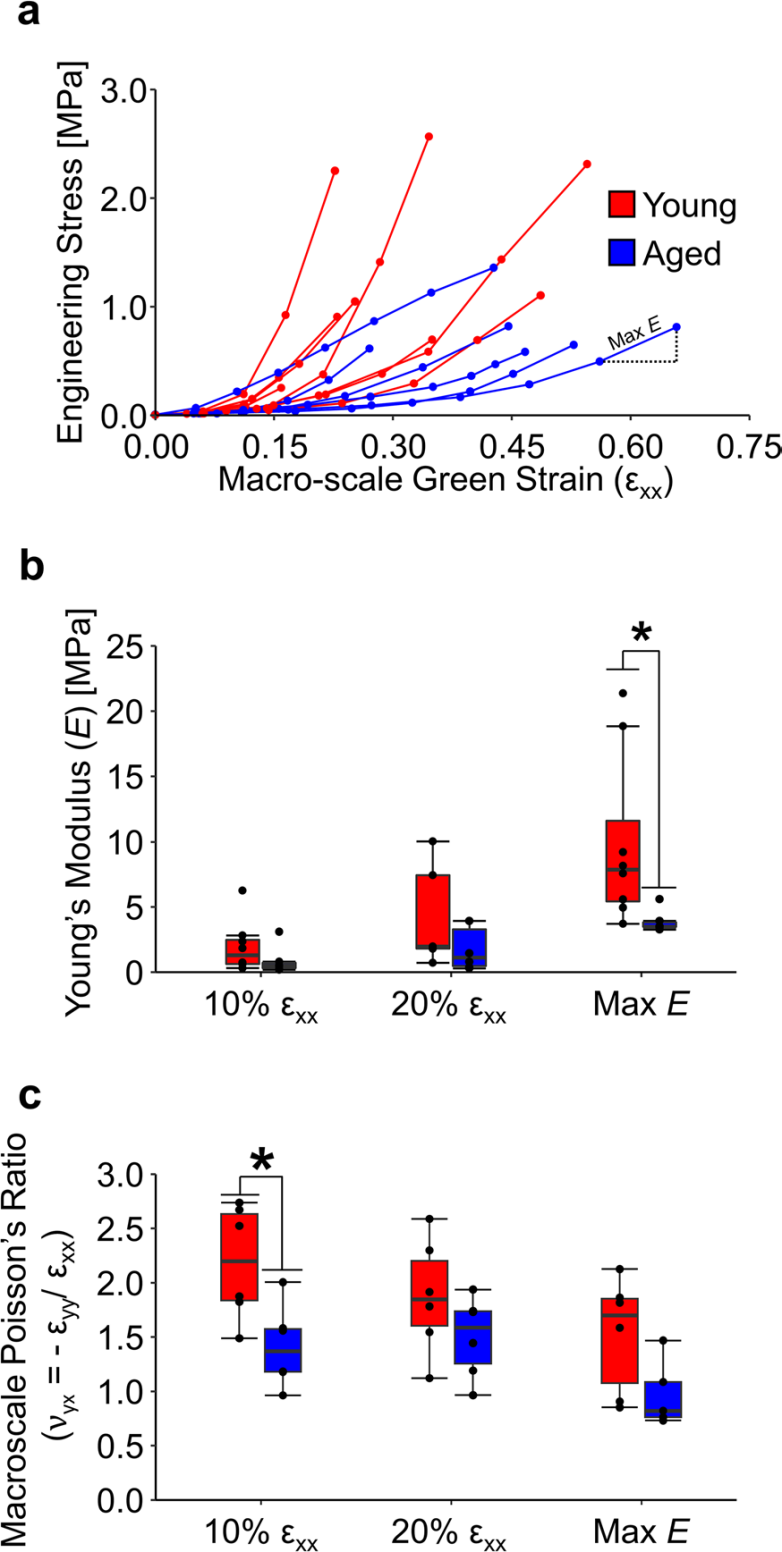
The macro-scale mechanical response during uniaxial tensile stretch indicates aged tissue is less stiff and has less inward contraction at low strains. (**A**) Stress–strain curves for each sample were generated from the forces at static equilibrium measured during imaging. (**B**) Aged tissue had a lower Young’s modulus than young tissue that was significantly different at peak stiffness (Max *E*). (**C**) At 10% strain, aged tissue had significantly less macro-scale contraction in the direction orthogonal to the imposed stretch. N = 6 for both age groups, * represents p < 0.05

Although the non-linear (i.e. toe) region of the stress–strain curves in both aged and young skin varied, aged skin reached lower peak stresses than young skin (Fig. 3A). When the Young’s modulus was quantified at 10% and 20% macro-scale Green strain, there was no significant difference between age groups (p ≥ 0.23). However, the maximum Young’s modulus for aged tissue (3.88 ± 0.87 MPa) was significantly lower than young tissue (9.93 ± 6.56 MPa; p = 0.04), suggesting that skin becomes less mechanically stiff with age (Fig. 3B). Furthermore, the Poisson’s ratio of aged tissue (1.41 ± 0.38) at 10% strain was significantly lower than young tissue (2.19 ± 0.52; p = 0.02), but not at 20% strain (p = 0.19) or maximum Young’s modulus (p = 0.06) (Fig. 3C).

### Micro-scale fiber and tissue kinematics

**Fig. 4:**
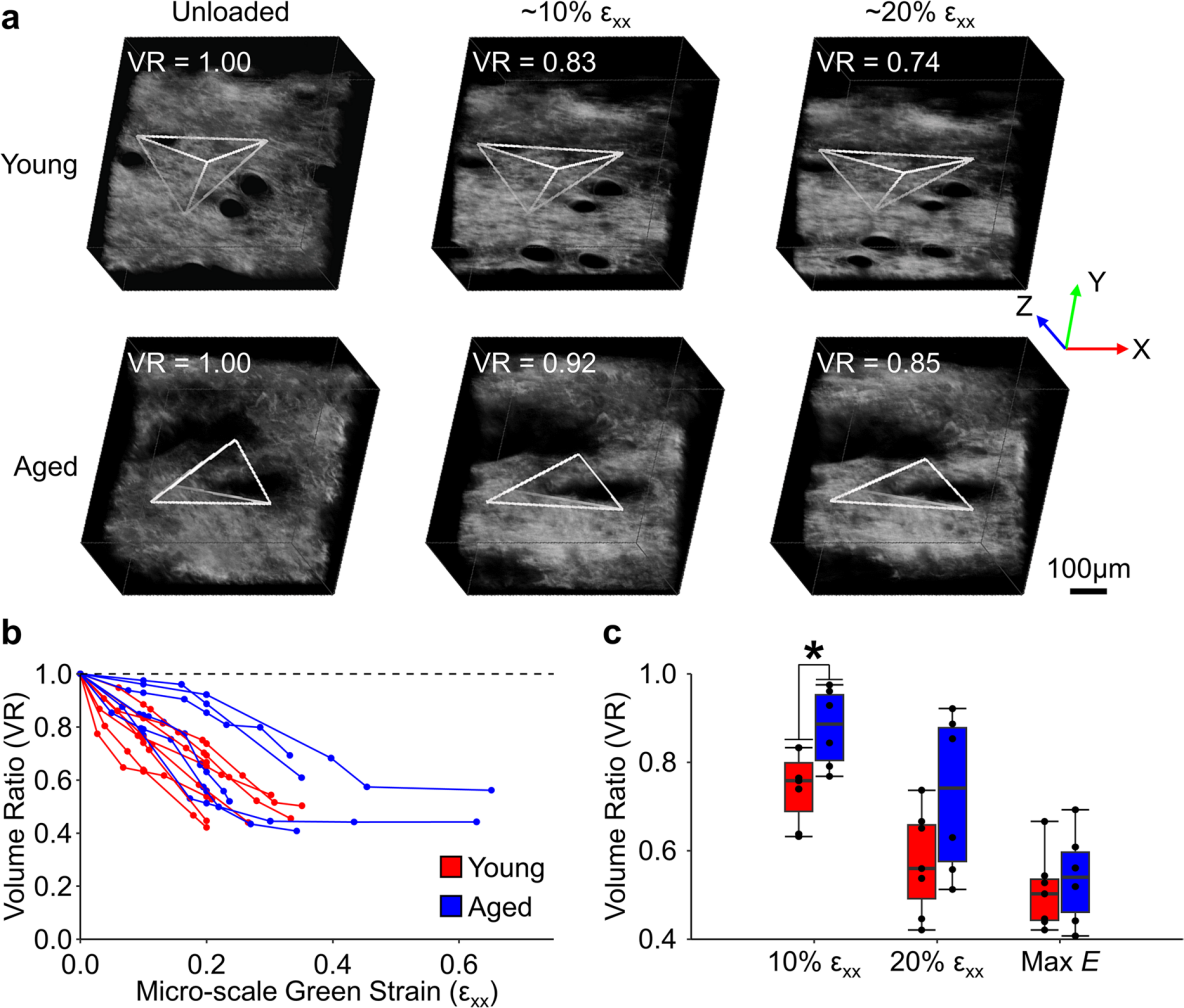
Micro-scale volume ratio was measured for young (**A; top**) and aged (**A; bottom**) tissue from features that were tracked within the SHG image volumes (white polygons). Scalebar represents 100 μm. A substantial decrease in volume ratio with increasing stretch was observed for both age groups. (**B**) Volume reduction was generally greater for young samples and significantly so for 10% micro-scale Green strain (ε_xx_) (**C**). N = 6 for both age groups, * represents p < 0.05

The age-related differences observed in the macroscale Poisson’s ratio of skin at low strain values were consistent with the microscale volume ratio (Fig. 4A). For both young and aged tissue, the volume ratio decreased with increasing stretch (Fig. 4B) [23]. Interestingly, at 10% strain, the tissue volume ratio of aged tissue (0.88 ± 0.09) was significantly larger than young tissue (0.74 ± 0.08; p = 0.02) (Fig. 4C). This indicates that during the initial stages of uniaxial stretch, aged skin does not compact in the orthogonal directions nearly as much as young skin. With increased strain, the volume ratio continued to decrease for both age groups, but the differences between groups became less significant at 20% strain (p = 0.11) and at the point of maximum Young’s modulus (p = 0.57) (Fig. 4B, C).

**Fig. 5:**
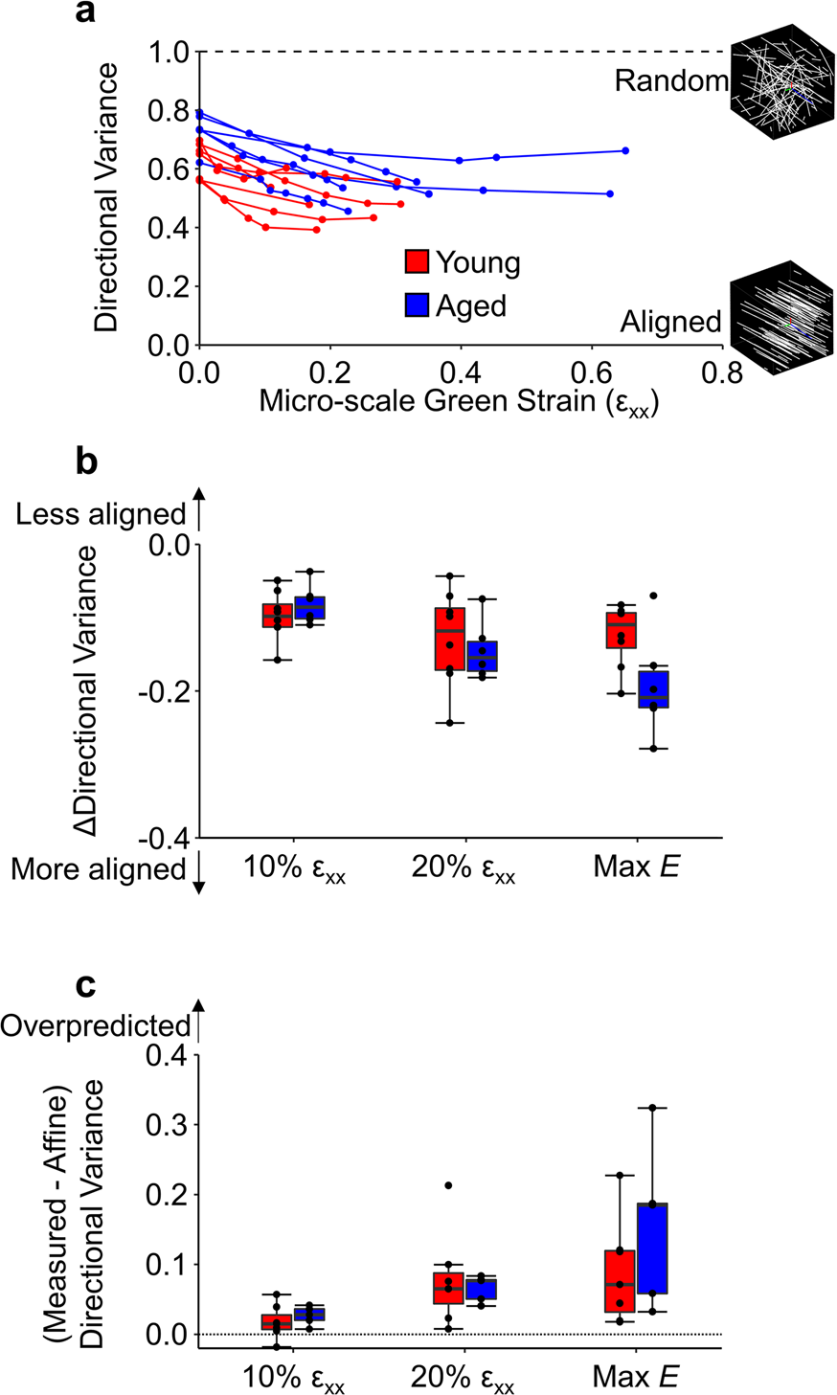
At each 1 mm increment of displacement, the 3D directional variance of collagen fibers was computed from all collagen-positive pixels within an image volume. During stretching, the collagen fibers in both age groups realigned towards the direction of stretch (**A**), and slightly greater realignment occurred in aged samples at maximum Young’s modulus (**B**). The measured realignment was also found to be significantly overpredicted when compared to theoretical realignment assuming an affine relationship with tissue deformation (**C**). N = 6 for both age groups

As skin was stretched, collagen fibers in both aged and young skin realigned toward the direction of loading resulting in a decrease in directional variance with increased strain (Fig. 5A). The 3D directional variance of aged skin was significantly higher than young skin in the undeformed configuration (Fig. 2C), and this relative difference between groups was preserved during uniaxial stretching. Thus, the level of fiber re-alignment during loading as measured by the change in directional variance was not significantly different between age groups (p > 0.07) (Fig. 5B).

In previous work, we showed that the measured 3D directional variance in young mouse skin does not share an affine relationship with the local tissue deformation [23], and in this study we confirmed this same result in aged skin. At 10% stain, 20% strain, and at maximum Young’s modulus, the difference between the measured and theoretical affine 3D directional variance was significantly greater than 0 (p < 0.003) (Fig. 5C). This indicates that the amount of fiber re-alignment is overpredicted when a theoretical affine assumption between 3D fiber kinematics and local tissue deformation is made for aged tissue as well. Finally, there were no statistical differences found between the magnitude of overprediction between aged and young tissue (p > 0.29).

## Discussion

Advanced aging is typically characterized by poorly organized collagen fibers and an increased amount of crosslinking, which results in altered mechanical properties [2, 5, 40]. In this study, we characterized the initial 3D microstructure of young and aged mouse skin, as well as quantified the 2D macro- and 3D micro-scale kinematic response due to uniaxial mechanical stretching. In an undeformed state, we saw both an increase in non-enzymatic crosslinks measured by two-photon excited fluorescence (Fig. 2A, E) and a reduction in fiber alignment measured by second harmonic generation imaging (Fig. 2C) with respect to intrinsic aging. During incremental uniaxial mechanical stretching, the maximum Young’s modulus of aged skin was significantly lower than that of young skin (Fig. 3A). This collection of age-related differences is consistent with previous work [2, 40–42], and our unique quantitative multi-scale analysis allowed us to characterize a variety of additional differences in the microstructural kinematics of aged and young skin. The 2D macro- and 3D micro-scale tissue kinematics of aged skin revealed a diminished response in the direction orthogonal to stretching within the first 10–20% strain, but then a similar tissue response was observed as skin reached maximum stiffness within the linear region of the stress–strain curve (Fig. 3C). Although collagen fibers were initially less aligned in aged skin (Fig. 2C), fibers in both age groups similarly rotated toward the direction of loading and caused a decrease in fiber directional variance with increased stretch (Fig. 5). This fiber realignment in the direction of stretch was less than expected if one assumes that the fiber kinematics are affine with the local deformation, but this finding is consistent with our previous observations [23].

The increase in crosslinking and more random fiber organization with increased age that was observed in this study may be related to changes in collagen fiber remodeling. Within the dermis, resident fibroblasts secrete matrix metalloproteinases (MMPs) that break down collagen fibers [43, 44], but non-enzymatic crosslinks are irreversible and cannot be digested by MMPs. This results in an accumulation of crosslinked fiber network fragments with age and a decrease in internal tension on the fiber network [41]. The decrease in mechanical tension negatively impacts new fiber deposition by fibroblasts, making the collagen fiber network more fragmented or less connected over time [29, 41]. Additionally, there is no difference in the amount of SHG signal from all collagen-positive pixels within an image volume (Fig. 2B), suggesting no significant age-related change in the ultrastructural organization within the collagen fibers.

Skin stiffness was reduced in aged skin (Fig. 3A), and we hypothesize this is related to the altered dermal microstructure through a decreased level of collagen fiber recruitment [14, 35, 45]. During uniaxial tensile loading, collagen fibers within a soft tissue typically become recruited into a stress-bearing role with increasing strain. The more random initial alignment of collagen fibers in aged skin will result in less fiber engagement, particularly during the toe region of the stress–strain curve, ultimately resulting in lower tissue stiffness if all fibers are not mechanically stretched prior to failure [45]. Reduced collagen fiber recruitment during tensile loading in aged skin may also explain the lower macro-scale Poisson’s ratio (Fig. 3B) and higher micro-scale volume ratios (Fig. 4C) that were measured at 10% strain. Multi-scale fiber network modeling of our previous experimental results involving young skin indicated that the volume reduction in skin during stretching is associated with the rotation of highly connected collagen fibers [14]. More recent simulations of the dermal microstructure indicate that network fragmentation due to aging can reduce fiber recruitment and lower tissue stiffness [46].

Collagen fibers within the dermis of skin form a dense, inter-woven structure that is different than the highly aligned and crimped fibers found in many other soft tissues such as tendon or heart valve [47]. During the toe region of the stress–strain curve, entangled collagen fibers become recruited to bear some of the applied load, causing them to slightly rotate toward the axis of stretch. This fiber rotation then causes the tissue to contract inward perpendicular to the direction of stretch [14]. However, aged tissue may not have the same magnitude of collagen rotation and recruitment as young tissue due to its more random organization and increased network fragmentation. This reduction in collagen entanglements could cause a reduction in inward contraction at both the macro- and micro-scale as observed in the current study (Fig. 3C and 4C). Of note, the volume ratio of aged tissue at 20% strain is similar to the volume ratio of young tissue at 10% strain, suggesting that as more collagen fibers in aged tissue are engaged by tensile loading, the mechanical response is similar to young tissue. In other words, the increased laxity of aged skin is likely associated with altered microstructural organization, which impacts fiber kinematics within the toe region, and lowers the tissue’s elastic modulus. Additionally, the change in 3D directional variance during stretching was not different between age groups, indicating that although collagen fibers can be recruited similarly during mechanical loading in both age groups, the fraction of recruited fibers is always less in aged skin (Fig. 5A, B) due to its different unloaded configuration. For both age groups, the amount of fiber realignment in the direction of loading was less than expected based on the assumption of affine fiber kinematics relative to the local 3D deformation. This is somewhat surprising, because other soft tissues like tendon are known to exhibit affine fiber kinematics at the microscale [47]. However, the collagen fibers of the dermis are more entangled and randomly oriented than they are in tendons, which likely results in more complex, non-affine fiber kinematics [19, 23]. Although more work is require to fully understand the collagen fiber kinematics of the dermis in response to loading, no significant age-related differences were observed in this non-affine behavior (Fig. 5C).

The relationship between skin structure and function, and how it changes with intrinsic aging, has been interrogated by many, but there is no clear consensus on how skin stiffness changes with age. During aging, skin undergoes many structural changes, such as an increase in collagen fragmentation, thinning, and a reduction in the amount of aligned collagen fibers [2, 40, 41]. These alterations in structure clearly impact its mechanical properties, but determining precisely how is, in part, compounded by differences in loading conditions and testing methods employed [2]. For example, in vivo mechanical testing regimes, such as torsion or suction testing, report an increase in stiffness with age, but the measured mechanical properties of skin from those devices require assumptions about the material properties and geometry of skin that may not be entirely correct [48]. Moreover, experiments utilizing uniaxial or biaxial mechanical testing measure either an increase or decrease in stiffness with age, and this discrepancy may be attributed to differences in the loading protocols, sample preparation, or anatomical location of the skin [49, 50]. For example, using similar mechanical testing conditions as the current study, Lynch et al. observed a significant increase in skin stiffness with age, but used depilated and de-epidermalized dorsal skin rather than intact skin from the ventral side of the torso [29]. Furthermore, when tissue was excised from a similar location by Lin, et al., mouse skin stiffness was found to decrease with age [42]. Interestingly, a recent study involving compressive mechanical testing of aged human skin suggests that the degraded dermal network leads to a more relaxed configuration that does not resist deformation, and therefore has decreased mechanical stiffness [51]. Overall, the lack of consensus on mechanical changes with age is likely related to differences in testing methods, sample preparation, loading conditions, the direction of loading, anatomical location, and the species [2, 42, 51, 52]. Microstructural models of mechanical function can be used to understand how different tests and experimental conditions may result in different age-related outcomes. However, our results indicate that skin does not respond to mechanical loads like many models of collagenous soft tissues. For example, skin significantly reduces in volume during tensile stretch, and its fiber kinematics are not affine with the local deformation. Therefore, the standardization of mechanical testing protocols and use of experimentally- informed computational models is critical for greater understanding the relationship between skin structure and function.

Finally, it is important to discuss the limitations of the imaging and biomechanical techniques used in this study. Although multiphoton microscopy allows for label-free imaging of collagen fibers and tissue autofluorescence, the depth of our imaging into the dermis is limited by the various scatterers and absorbers found in skin. For our experimental setup, we can consistently collect data within the first 200 µm of the skin, which is well within the reticular dermis of the tissue [52]. However, approaches capable of imaging deeper within the dermis may provide additional insights on the relationship between the microstructure and overall mechanical function of skin. Additionally, the uniaxial testing conditions used in this study result in unconstrained deformation orthogonal to the direction of stretch. We and others [21–23] have reported substantially large Poisson’s ratios for these loading conditions, but rigorous multi-axial mechanical testing devices may be needed to fully characterize the mechanical behavior of this planar tissue. Our study did not characterize all key constituents of skin. Elastin is well known for its contribution to the structure and function of soft tissues, and is known to decrease in skin with advanced age [2, 53]. However, mouse skin has substantially fewer elastin fibers than human skin, and those elastin fibers present are known to be substantially thinner [29]. Finally, although trends between stiffness and age were observed, the nominal values of stiffness measured in this study must carefully be considered due to the stress relaxation occurring during incremental mechanical testing. Additional work is required to understand how dynamic loading at high strain rates may result in different age-related outcomes compared to the quasi-static mechanical behavior measured in the current study.

## Conclusion

This study used non-destructive label-free multiphoton microscopy to investigate how the 3D dermal microstructure of skin changes during intrinsic aging, and how these changes are associated with mechanical function. Multiphoton microscopy has been used in the past during mechanical testing of aged skin, but this study uniquely reports on the 3D deformation and fiber kinematics of the dermis. The mechanical response of skin is highly dependent on the direction(s) of loading, fiber organization in that direction, and fiber-level properties like degree of crosslinking. Traditionally, the altered mechanical behavior in aged skin has been attributed to processes such as non-enzymatic crosslink fragment accumulation, decreased collagen fiber content, and morphological changes (i.e. thinning of the dermal layer) [5, 29, 54]. Our findings suggest that the micro-scale fiber organization and kinematics play a critical role in the changes in mechanical function of skin with advanced age. This detailed assessment of 3D fiber and tissue kinematics during uniaxial tensile loading lays the groundwork for future experiments which can expand these kinematic investigations to more complex loading scenarios.

## Data Availability

The raw data supporting the conclusions of this article are available from the corresponding author upon reasonable request.

## Abbreviations

SHG: Second harmonic generation
TPEF: Two-photon excited fluorescence
CNN: Convolutional neural network
